# Viral Load Distribution in SARS Outbreak

**DOI:** 10.3201/eid1112.040949

**Published:** 2005-12

**Authors:** Chung-Ming Chu, Vincent C.C. Cheng, Ivan F.N. Hung, Kin-Sang Chan, Bone S.F. Tang, Thomas H.F. Tsang, Kwok-Hung Chan, Kwok-Yung Yuen

**Affiliations:** * United Christian Hospital, Hong Kong Special Administrative Region, People's Republic of China; † University of Hong Kong, Hong Kong Special Administrative Region, People's Republic of China; ‡ Department of Health, Hong Kong Special Administrative Region, People's Republic of China

**Keywords:** SARS, transmission, viral load, Amoy Gardens, research

## Abstract

An unprecedented community outbreak of severe acute respiratory syndrome (SARS) occurred in the Amoy Gardens, a high-rise residential complex in Hong Kong. Droplet, air, contaminated fomites, and rodent pests have been proposed to be mechanisms for transmitting SARS in a short period. We studied nasopharyngeal viral load of SARS patients on admission and their geographic distribution. Higher nasopharyngeal viral load was found in patients living in adjacent units of the same block inhabited by the index patient, while a lower but detectable nasopharyngeal viral load was found in patients living further away from the index patient. This pattern of nasopharyngeal viral load suggested that airborne transmission played an important part in this outbreak in Hong Kong. Contaminated fomites and rodent pests may have also played a role.

## Abstract 2

Airborne transmission may have resulted in an outbreak of SARS in Hong Kong.

Severe acute respiratory syndrome (SARS) is a rapidly progressive pneumonia that affects all age groups in an epidemic manner. The number of cases worldwide has reached >8,000 with 774 deaths within a period of 9 months (*1*). A community outbreak affected 321 residents of a densely populated housing estate, the Amoy Gardens in Hong Kong, from March 20 to April 15, 2003 (*2*). This housing estate consists of 19 high-rise apartment blocks (A–S). Each block has 33 floors and 8 units per floor. Residents from 15 blocks were affected. The mechanism of the spread of SARS in Amoy Gardens has remained enigmatic. The suggestion has been made that virus-laden aerosols were forced from the sewage system by negative pressure of an exhaust fan in an airshaft into the dried U trap of the toilet in the bathroom of the index patient (*3*). Results of another study in which computer modeling was carried out without virologic proof suggested that these contaminated aerosols were spread by natural air currents to other apartment units (*4*). Other means of spread might have been droplet transmission among residents or by rodent pests (*5*).

In a recent study, mice experiments demonstrated that viral load in respiratory specimens was proportional to viral inocula in patients infected with SARS-associated coronavirus (SARS-CoV) (*6*). We hypothesized that the initial nasopharyngeal viral load would be higher in patients residing near the index patient and lower in patients living further from the index patient. We analyzed the distribution of the initial SARS-CoV viral load by quantitative reverse transcription–polymerase chain reaction (RT-PCR) of nasopharyngeal aspirates of the first 79 SARS patients from Amoy Gardens admitted to our hospital. We also correlated the pattern of viral load with the geographic distribution of these patients from Amoy Gardens, which may indicate the mode of transmission in this point-source outbreak.

## Patients and Methods

**Figure 1:**
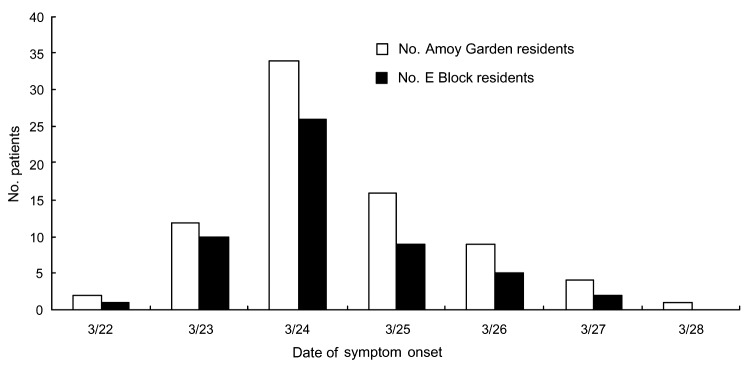
Numbers of patients in the initial outbreak of severe acute respiratory syndrome in Amoy Gardens admitted to United Christian Hospital, Hong Kong, 2003. The index patient visited Amoy Gardens on March 14 and March 19, 2003.

From March 24 to March 29, 2003, the first 79 SARS patients who lived at Amoy Gardens were admitted to the United Christian Hospital in Hong Kong (Figure 1). Since Amoy Gardens was placed under active surveillance by the health authority soon after the first few cases of SARS were detected, these patients underwent frequent examinations and were admitted early in the course of their illness (*7*). Their initial clinical signs and symptoms and progress have been previously reported (*7*). We prospectively collected demographic, clinical, and laboratory data from these first 79 SARS patients from Amoy Gardens who were admitted to the hospital. The diagnosis of SARS was confirmed by World Health Organization clinical and laboratory diagnostic criteria. SARS was defined clinically by fever (temperature >38°C), cough or shortness of breath, and new pulmonary infiltrates on chest radiographs or by high-resolution computed tomographic scans in the absence of an alternative diagnosis to explain the clinical manifestations. Positive SARS diagnostic findings included at least 1 of the following: confirmation by a positive PCR result for SARS-CoV, seroconversion by enzyme-linked immunosorbent assay or immunofluorescent antibody assay, or virus isolation in cell culture plus PCR confirmation (*7*).

**Figure 2:**
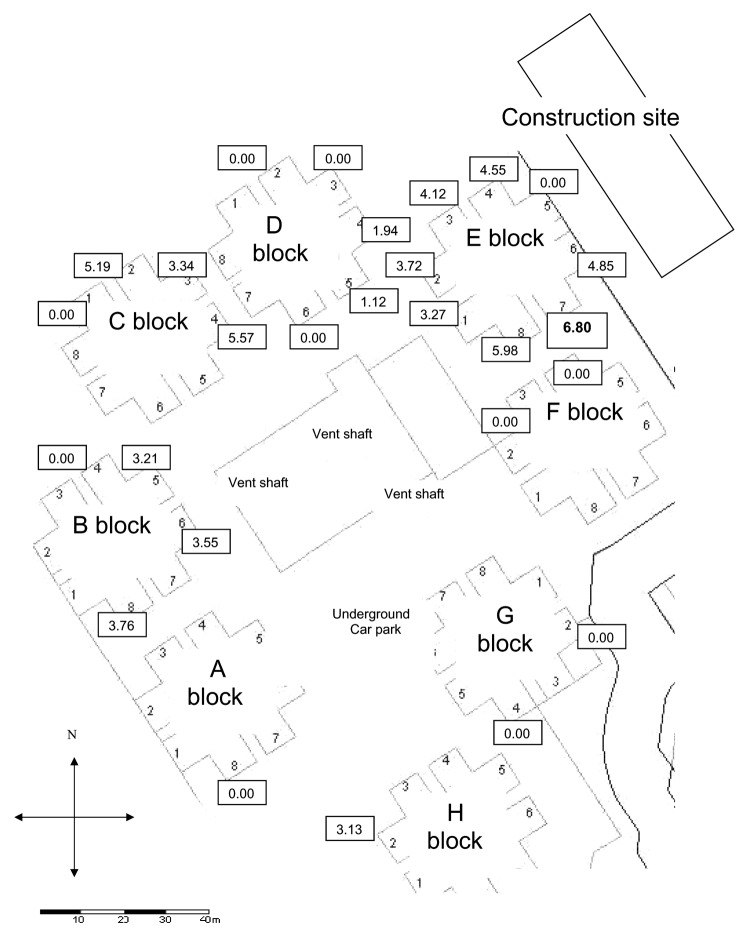
Scaled map of Amoy Gardens units and distribution of the median viral load (log10 copies/mL) of the nasopharyngeal specimens (values in boxes) of patients in their respective residential blocks (index patient lived in E7).

Each apartment unit was coded according to block (A–H) and unit (*1**–**8*) (Figure 2). Patients in 26 different unit codes were affected. We retrospectively studied the viral loads of the first nasopharyngeal aspirate taken on the day of admission of the SARS patients who were admitted within the first 6 days of the epidemic. We examined the relationship between the viral loads and the distribution of the patients in Amoy Gardens. The index patient, who was responsible for transmitting the disease, stayed for 2 days (March 14 and 19, 2003) in block E unit 7 (E7, floor 16) and infected his brother (*4*), our first patient. The distance of the different block units from E7 was measured (Figure 2). Viral load was measured as previously described (*7**,**8*).

We compared the clinical characteristics and nasopharyngeal viral load of these patients in different blocks by chi-square test for categorical variables, Student t-test, or Mann-Whitney U test for continuous variables where appropriate. Correlation of nasopharyngeal viral loads in relation to the distance from the index patient was calculated by Spearman correlation. The patients were categorized into 5 subgroups according to the distance from the block of the index patient for further analyses: block E7, block E other than E7, blocks D and F, blocks C and G, and blocks A, B, and H. All statistical analyses were performed with SPSS version 12.0 software (SPSS Inc., Chicago, IL, USA). A 2-tailed p value <0.05 was considered significant.

## Results

The number of patients in the initial outbreak of SARS in Amoy Gardens is shown in Figure 1. The demographic, clinical, and laboratory characteristics of patients residing in E block (where the index patient resided) and those residing in non-E blocks were compared (Table). Seventy-five patients (94.9%) were Chinese and 4 were Filipino. There were 38 male and 41 female patients. The mean (SD) age was 39.4 (11.5) years (range 20–72 years). Fifty-three patients (67.1%) were residents of E block; 10 (12.7%) were residents of E7 and 25 (31.6%) were residents of E8.

**Table:** Demographic factors on admission of patients living in E block and non-E block of Amoy Gardens, Hong Kong*

Factor	E block patients (n = 53)	Non-E block patients (n = 26)	p value
Age, y, mean (SD)	40.6 (11.9)	37.1 (10.6)	0.21†
Male:female ratio	25:28	13:13	0.81‡
Duration of symptoms to admission (days), mean (SD)	2.4 (1.2)	2.5 (1.2)	0.72†
Coexisting conditions including chronic hepatitis B, no. (%)	11 (19.6)	8 (30.8)	0.33‡
Chronic hepatitis B infection, no. (%)	5 (9.4)	5 (19.2)	0.22‡
Abnormal chest radiograph results, no. (%)	38 (71.7)	21 (80.8)	0.38‡
Multilobar involvement on initial chest radiograph, no. (%)	13 (24.5)	5 (19.2)	0.60‡
Day of collection of nasopharyngeal specimens after onset of symptoms, mean (SD)	3.2 (1.2)	3.3 (1.8)	0.74†
Quantitative RT-PCR result of nasopharyngeal specimens (log_10_ copies/mL), median (IQR)	5.09 (3.50–6.59)	0 (0–3.57)	0.008§
Hemoglobin (g/dL), mean (SD)	13.3 (1.7)	13.8 (1.4)	0.24†
Neutrophil count (× 10^9^/L), mean (SD)	5.2 (2.0)	5.3 (2.0)	0.77†
Lymphocyte count (× 10^9^/L), mean (SD)	0.92 (0.6)	0.86 (0.4)	0.65†
Sodium (mmol/L), mean (SD)	138 (3)	138 (3)	0.91†
Potassium (mmol/L), mean (SD)	3.9 (3.6)	3.9 (3.0)	0.31†
Urea (mmol/L), mean (SD)	4.4 (1.4)	4.1 (1.0)	0.34†
Creatinine (μmol/L), mean (SD)	88 (16)	85 (12)	0.35†
Alanine aminotransferase (IU/L), mean (SD)	41 (49)	31 (19)	0.31†
Albumin (g/L), mean (SD)	40 (3)	40 (3)	0.85†
Creatinine kinase (IU/L), mean (SD)	197 (222)	190 (186)	0.89†
Lactate dehydrogenase (IU/L), mean (SD)	437 (190)	398 (107)	0.47†

*SD, standard deviation; IQR, interquartile ratio; RT-PCR, reverse transcription–polymerase chain reaction.

†By Student *t* test.

‡By chi-square test.

§By Mann-Whitney U test.

**Figure 3:**
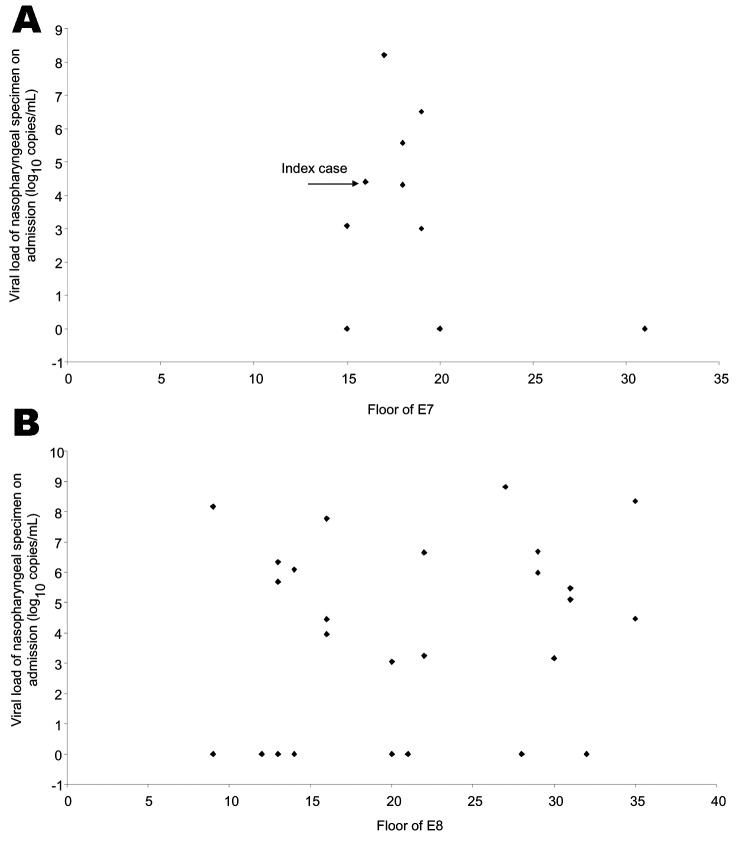
Distribution of viral load in nasopharyngeal specimens (log10 copies/mL) of Amoy Gardens residents in E7 (A) and E8 (B).

The relationships between viral load and distribution of patients from E7 and E8 are shown in Figure 3A and B, respectively. In E7, patients who resided within a few stories of the 16th floor had higher viral loads. For cases in neighboring E8, the distribution of patients and viral loads was random.

The median nasopharyngeal viral load in E block patients (5.09 log_10_ copies/mL) was much higher than in non-E block patients (0 log_10_ copies/mL) on admission (p<0.001). On admission, no statistically significant differences were found between E block patients and non-E block patients in terms of all demographic characteristics, initial radiographic findings, and baseline laboratory results (Table). The mean day of collection of nasopharyngeal specimens from E-block and non-E block patients did not differ significantly. Overall, the mean (SD) number of days from onset of symptoms to collection of nasopharyngeal samples was 3.22 (1.5), and no correlation was found between initial nasopharyngeal viral load and time elapsed from symptom onset date to the day of sample collection (Spearman ρ –0.16, p = 0.156).

**Figure 4:**
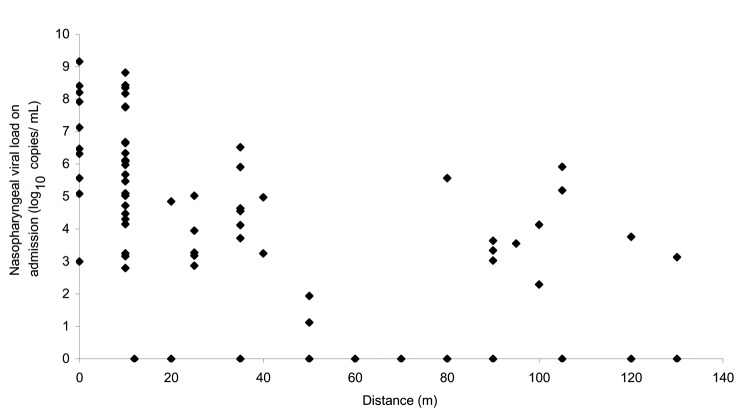
Correlation of nasopharyngeal viral load (log10 copies/mL) in relation to the distance from the index unit (E7).

Median viral loads of each unit of different blocks are shown in Figure 2. The initial nasopharyngeal load of patients was highly correlated with the distance in relation to the block of the index patient (Spearman rho –0.63, p<0.001, Figure 4). The percentage of specimens with a negative nasopharyngeal viral load in each block in order of patient distance from block E was as follows: block E (4/52) 7.7%; block D (4/7) 57.1%; block F (2/2) 100%; block C (1/6) 15.2%; block G (2/2) 100%; block A (1/1) 100%; block B (2/6) 33.3%; and block H (1/1) 100% (p = 0.04 by chi-square test). Subgroup analysis showed that patients in E7 and E8 had the highest median viral load, 6.80 and 5.98 log_10_ copies/mL, respectively. Patients from these 2 units also accounted for 12.7% and 31.6% of the total number of patients, respectively. This pattern of distribution is strongly affected by the distance of the patients' units from the index patient (Figures 2 and 4). On the basis of a visual inspection of the layout of the units (Figure 2), the direction in which patients' flats faced may also have influenced the viral load; patients in flats that faced away from the index patient's unit had a lower viral load.

The overall case death rate among the 79 patients was 24.1%. The highest rate was in block E, which accounted for 79% of all deaths, while the death rate in patients living in E7 (the same block as the index patient) was 70% (7 patients). This rate is significantly higher than in other units (p = 0.001 by χ^2^ test). The index patient was one of the few patients from E7 who survived the disease.

## Discussion

In this study, a higher viral load was observed in patients who lived near the index patient than in those who lived further away. Amoy Gardens was placed under active surveillance during the SARS outbreak and the residents underwent frequent examinations. They were admitted to the hospital soon after any symptom of SARS developed, and nasopharyngeal specimens were collected at an early stage. Variation in collection time cannot explain the viral load distribution. The size of the viral inocula may have progressively decreased downstream. A recent study has demonstrated in mice that SARS-CoV viral load in the respiratory tract is proportional to viral inocula administered intranasally (*6*). Similarly, the degree of viremia is related to the size of the viral inoculum in HIV and hepatitis C virus infections in various models (*9**,**10*). Three patients from E7 and 12 patients from E8 had a higher viral load and more severe disease than the index patient in E7 (Figure 3). This finding can be explained by the fact that secondary case-patients had probably received higher viral inocula through droplets or close contact (*11**–**14*). The viral load gradually decreased in tertiary patients who lived further from the index case; dilution factors may have had an effect. Moreover, the shape of the U trap and the warm aerosol generated from the bathroom of the index patient caused the aerosol to circulate upwards, which may explain why only the upper floors of E7 were initially affected. Subsequently the virus-laden aerosols cooled and sank. At the same time, the virus was carried by a southwestward wind to E8 and other parts of Amoy Gardens. This scenario accounted for the higher rate and more widespread distribution of SARS patients in E8 than E7.

How SARS is transmitted is variously explained. In most cases, SARS is transmitted by direct contact with ill persons and spread of large droplets (*15*). In more distant transmission, airborne spread, contaminated fomites, and rodent pests can spread this disease (*4**,**5**,**16*). The initial viral load pattern in our study may help explain the different mechanisms involved in transmitting SARS in this outbreak. The highest nasopharyngeal viral load was detected in patients residing in E7 and E8, which were near the unit inhabited by the index patient. Direct contact transmission with the index patient and droplet spread by cough may have occurred among patients living in block E. Rodent pests may have spread the virus in the same block or even to distant blocks. Transmission by contaminated fomites such as elevator door knobs or door handles would also lead to spread among patients in the same block.

The viral load of each patient correlated with the distance in relation to the index block (E7). However, more patients and higher viral load were found in patients living in block D than block F, in block C than block G, and in block B than blocks A and H, even though they were a similar distance from the index block (Figure 2). The attack rate was highest in block E, which accounted for 41% of the 321 SARS cases in Amoy Gardens, followed by block C (15%), block B (13%), and block D (13%). The remaining cases (18%) were distributed in 11 other blocks (*2*). This distribution pattern can be explained by airborne transmission as virus-laden aerosols circulated inside the complex and were driven by a southwestward wind from block E to blocks D, C, and B (*4*). Meteorologic data from The Hong Kong Observatory, Hong Kong Special Administrative Region showed that the prevailing wind direction on March 14 and March 19, 2003 was from the southwest. This pattern is consistent with a hypothesis of airborne transmission (*17*). Patients living in block D had a lower viral load than those in blocks B and C, even though they lived closest to the index patient. They may have been protected by a nearby construction site (Figure 2), which created a shield against the virus-laden draft. Patients from E7 living on floors 15–20 had higher viral loads than those living above or below them (Figure 3A). This distribution may be the result of a dilution effect as the virus-laden plume rose from the middle floors to the higher floors. Nonetheless, the airborne hypothesis is not possible to prove because simultaneous air sampling and analysis of the SARS viral load was not carried out.

Severity of illness did not differ between block E patients and non-E block patients when they were first seen at the hospital, despite higher viral load in block E patients. However, the death rate was higher in block E. We have previously demonstrated that patients with high initial and peak viral loads in nasopharyngeal samples were more likely to show a less favorable disease course and lower survival rate (*8**,**18*). Patients living in E7 who had highest nasopharyngeal viral loads explains why their death rate was higher than for those living in other units. The dilution effect resulted in a decreased viral load as the disease spread to other units and in a lower death rate.

Our study was limited because we analyzed only data on the first 79 of 321 patients in Amoy Gardens with SARS. This limitation was the result of the rapid influx of patients who overwhelmed the capacity of our hospital; additional patients were admitted to other hospitals for treatment. Second, no human study has confirmed the relationship between the size of viral inocula and viral load. Host factors are important in this regard (*9*). Nevertheless, we believe that the patients we studied provide important information regarding initial viral loads and geographic factors. This situation involved different modes of transmission, including direct contact, droplets, airborne, contaminated fomites, and rodent pests. No single mechanism could explain such a major outbreak.

In conclusion, the overcrowded housing complex, unconnected pipes, a southwestward wind, rodent pests, and arrival of the SARS index patient all created an environment favorable for the transmission of this disease. Different modes of transmissions apparently had a part in this major outbreak. What actually took place will likely remain unsolved. Nevertheless, the possibilities of different modes of spread alert us to the importance of a multicomponent infection control policy in future outbreaks of SARS-CoV infection, as well as in other respiratory viral infections.
